# Supplementary material for: Prevalence, spatial patterns and determinants of zero-dose vaccination among children aged 12–23 months in Sub-Saharan Africa: A multilevel spatial analysis
Source: PLoS One. 2026 Mar 20;21(3):e0344293. doi: 10.1371/journal.pone.0344293 (PMC13004531; doi:10.1371/journal.pone.0344293)
Supplement: S1 File — (DOCX) [file pone.0344293.s001.docx]

**Data Management and Analysis**

For the descriptive analysis, data preparation and cleaning were carried out in STATA version 17 and Microsoft Excel 2021. The main outcome variable, zero-dose vaccination, was cross-tabulated with the cluster variable (v001). The resulting dataset was exported to Excel, where proportions of zero-dose vaccination were calculated. These proportions were saved as comma-separated value (CSV) files and later imported into ArcGIS 10.8, where they were linked to cluster coordinates. Clusters without corresponding geographic information were excluded. To create a unified dataset, country-specific CSV files containing zero-dose vaccination proportions were merged in Excel, with new cluster identifiers generated to replace the original ones. The combined dataset was then saved as a CSV file and imported into ArcGIS 10.8 for mapping and examining the spatial variation of newborn deaths within 24 hours of birth.

In STATA version 17, survey data were set up using the command svyset v021 [pw=v005], strata (v022). Here, v021 represents the primary sampling unit, v005 the sampling weight, and v022 the stratification variable. Applying svyset ensured that the analysis properly accounted for the complex survey design, producing accurate standard errors, confidence intervals, and p-values. It also adjusted for unequal selection probabilities and non-response using survey weights. All subsequent statistical were therefore based on the weighted sample.

For the multilevel spatial analysis, cleaned country datasets were first appended sequentially in STATA 17. GPS datasets containing latitude and longitude coordinates were also merged step by step, matched by survey cluster (v001) and country code (v000). The pooled GPS dataset was then integrated with the combined children’s records so that each child could be spatially referenced to its correct enumeration area. After merging, additional data cleaning and recoding were performed. The finalized dataset was then imported into R using the read_dta() function from the haven package, which allows direct import of Stata files. To ensure compatibility with the spatial delta-generalized linear mixed modeling (sdmTMB) framework, categorical predictors were converted into factors using the as.factor() function, while continuous variables such as the cluster number (v001) and sampling weight (v005) were converted into numeric format using as.numeric(). A unique cluster-country grouping variable was created by combining v001 and v000 with the mutate () function from dplyr, ensuring that random intercepts were correctly specified at the cluster level. Geographic coordinates (longitude and latitude) for each enumeration area were then used to construct a spatial mesh. With the make_mesh() function from sdmTMB, a triangulated irregular network (TIN) mesh was generated using 5,000 knots via a k-means clustering algorithm. This mesh discretized the spatial domain of sub-Saharan Africa and enabled implementation of the Stochastic Partial Differential Equation (SPDE) approach to model spatially structured random effects. To adjust for the complex survey design and unequal sampling probabilities, survey weights (v005) were applied during estimation. Finally, zero-dose vaccination was modeled as a binary under a binomial distribution with a logit link. This specification allowed the model to capture both individual- and cluster-level influences while accounting for spatial dependence across the study region.

**Comparison of Kriging techniques and Models**

Following comparison of several Kriging interpolation techniques, we found that Ordinary type was preferred becouse of its lowest Root-Mean-Square Standardized Error (RMSSE) of 1.02. In addition, the evaluation of the semivariogram models using key prediction performance metrics indicated that the Spherical model delivered the better predictive performance with its mean standardized error (MSE) of 0.01, reflecting minimal prediction bias and its RMSSE of 1.02 is nearly ideal, demonstrating well-calibrated uncertainty estimation. Therefore, this model provides the optimal balance of accuracy, uncertainty calibration, and precision, establishing it as the most suitable choice for Kriging (Table 4).

Suplimentary Table 1: Comparison of kriging techniques and semi-variogram models

| **Kriging Techniques** | **MSE** | | **RMSSE** | |
| --- | --- | --- | --- | --- |
| Ordinary Kriging | -0.0002 | | 1.02 | |
| Simple Kriging | -0.09488 | | 1.22 | |
| Universal Kriging | -0.0002 | | 1.03 | |
| Indicator Kriging | 0.0028 | | 1.16 | |
| Probability Kriging | 0.0142 | | 1.33 | |
| Disjunctive Kriging | 0.1627 | | 1.26 | |
| **Prediction Error** | **Circular** | **Spherical** | **Exponential** | **Gaussian** |
| **MSE** | 0.02 | 0.01 | 0.01 | 0.02 |
| **RMSSE** | 1.03 | 1.02 | 1.17 | 0.97 |

MSE: Mean Standardized Error, RMSSE: Root-Mean-Square Standardized Error
